# Inflammatory Treatment Used to Mimic Osteoarthritis and Patients’ Synovial Fluid Have Divergent Molecular Impact on Chondrocytes In Vitro

**DOI:** 10.3390/ijms24032625

**Published:** 2023-01-30

**Authors:** Enrico Ragni, Paola De Luca, Federico Valli, Luigi Zagra, Laura de Girolamo

**Affiliations:** 1 Laboratorio di Biotecnologie Applicate all’Ortopedia, IRCCS Istituto Ortopedico Galeazzi, Via R. Galeazzi 4, I-20161 Milano, Italy; 2 Chirurgia Articolare Sostitutiva e Chirurgia Ortopedica (CASCO), IRCCS Istituto Ortopedico Galeazzi, Via R. Galeazzi 4, I-20161 Milano, Italy; 3 Hip Department, IRCCS Istituto Ortopedico Galeazzi, Via R. Galeazzi 4, I-20161 Milano, Italy

**Keywords:** osteoarthritis, gene expression, inflammation, synovial fluid, extracellular matrix, cytokines, chondrocytes

## Abstract

Osteoarthritis (OA) is a chronic disease characterized by joint tissue disruption and inflammation with a paucity of therapeutic options. Chondrocyte in vitro models are commonly used as the first step in evaluating new approaches and rely on the stimulation of an OA-like phenotype with inflammation often the method of choice. Inflammatory priming is frequently based on cytokines used at concentrations very far from the reality in the patients’ synovial fluid (SF). The aim of this work was to compare the transcriptional response of chondrocytes to different inflammatory conditions: the high levels of IL1β that are used for standardized inflammation protocols, OA-SF, IL1β, IL6 and IFNγ at SF-like concentrations both individually and simultaneously to mimic a simplified “in vitro” SF. Both high IL1β and OA-SF strongly influenced chondrocytes, while SF-like concentrations of cytokines gave weak (IL1β alone or in combination) or no (IL6 and IFNγ alone) outcomes. Chondrocytes under the two most powerful polarizing conditions had a clearly distinct fingerprint, with only a shared albeit molecularly divergent effect on ECM stability, with IL1β mainly acting on ECM degrading enzymes and OA-SF accounting for a higher turnover in favor of fibrous collagens. Moreover, OA-SF did not induce the inflammatory response observed with IL1β. In conclusion, although partially similar in the endpoint phenotype, this work intends to encourage reflection on the robustness of inflammation-based in vitro OA models for molecular studies on chondrocytes.

## 1. Introduction

Osteoarthritis (OA) is a chronic disease currently affecting more than 500 million people worldwide. It is a whole joint disease that involves the articular tissues such as subchondral bone, synovium and cartilage, and includes meniscal degeneration and inflammation and fibrosis of the infrapatellar fat pad [1]. Alterations in mechanical, inflammatory and metabolic factors lead to structural destruction with changes in cartilage composition and the eventual loss of tissue integrity [2]. OA chondrocytes generate extracellular matrix (ECM) degradation products and pro-inflammatory mediators that act on the synovium stimulating the further increase in pro-inflammatory responses [3]. As a consequence, several pro-inflammatory factors characterize the fingerprint of the synovial fluid (SF) in OA patients [4], including, among others, the well-characterized IL1β (interleukin 1β), IFNγ (interferon γ), TNFα (tumor necrosis factor α) and IL6 (interleukin 6) [5].

Several drugs [6] and biological products [7] are actively sifted as future OA drugs, relying on in silico and in vitro models as the first step to test therapeutic activities [8]. For these reasons, validated in vitro cellular models are crucial. Two-dimensions (2D) in vitro models have historically been the most used for cytokine stimulation (i.e., IL1β to induce an OA phenotype on chondrocytes) and in turn to screen new chondroprotective drugs or biologics to attenuate the catabolic factors involved in cartilage degradation. One straightforward example is a recently published work that showed the effect of the flavonoid isoliquiritigenin on IL1β-induced production of matrix metalloproteinase and nuclear factor kB in rat chondrocytes [9], with in vitro results nicely anticipating those observed in vivo. The potential of this methodology in the screening of new compounds was recently confirmed by the observed chondroprotective effects of bone marrow mesenchymal stromal cells-derived extracellular vesicles (EVs) used as a cell-free approach on IL1β-stimulated chondrocytes obtained from OA patients [10]. Together with their clear advantages, 2D models are used to evaluate compound potency, also present are drawbacks that are inherent in the nature of the system itself. If on one side monolayer cultures are easily handled to manipulate gene and protein expression, on the other side extensively expanded chondrocytes may undergo de-differentiation and lose their distinct phenotype [11]. Additionally, three-dimensional cell organization and interaction with other counterparts are missing. Nevertheless, the net of pros and cons, 2D chondrocyte cultures still represent the first and easiest step in evaluating new compounds and drugs.

However, in this context, an often underestimated issue is the inflammatory stimulus used to mimic the OA environment. There are several models to induce the OA phenotype, ranging from the use of inflammatory cytokines such as IL1β, TNFα, IFNγ, or IL6 [12] or more complex stimuli such as conditioned medium from activated macrophages [13]. Due to its ease of application, the most used cytokine is IL1β, usually at a concentration (1 to 10 ng/mL) that is very far from the values detected in the SF of OA patients (often between 1 and 20 pg/mL) [14]. Of note, in several studies it was demonstrated that the chondrocyte response is directly dependent on IL1β concentration [15,16], and that at levels close to those in SF, chondrocyte feedback may be weak and/or undetectable for specific factors [17]. This is the reason why only in a few reports IL1β at 1–10 ng/mL is correctly described as an acute insult mimicking OA, with this issue neglected and high levels of cytokine proposed to represent a bona fide OA environment. Moreover, the use of a single molecule is a simplification of the definitely more complex scenario represented by the naïve OA synovial fluid, where several actors play a concerted role in tissues and cells, and the combination of at least a few factors could be considered to represent a situation closer to reality. For these reasons, in this report, the effects of naïve OA-SF, IL1β at 1 ng/mL and IL1β/IFNγ/IL6 at the concentration found in OA-SF, either individually or simultaneously, were assessed in chondrocytes by sifting the expression levels of several genes involved in inflammation and extracellular matrix homeostasis in OA. The purpose of this work is to shed light on the similarities and differences between current practice and a condition closer to OA in the frame of a 2D in vitro model.

## 2. Results

### 2.1. Flow Cytometry Analysis of Isolated Chondrocytes

**Figure 1:**
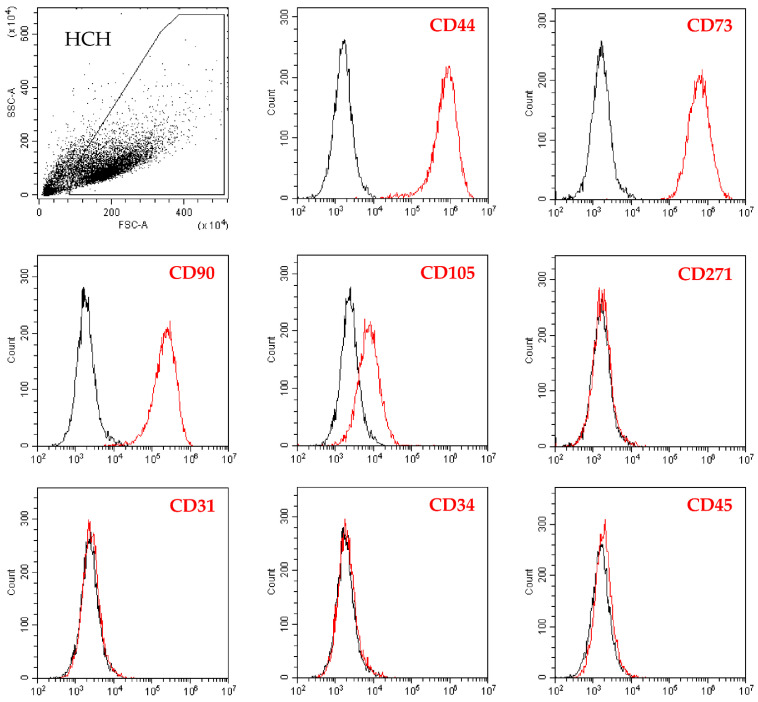
Flow cytometry analysis of human chondrocytes (HCH). After the identification of single cells by exclusion of debris (upper-left panel), staining for chondrocyte (CD44, CD73, CD90 and CD105) markers confirmed cell identity. Bone marrow stromal cell-specific (CD271) marker resulted negative, confirming absence of subchondral bone marrow contamination. Absence of hemato-endothelial (CD31, CD34, and CD45) markers staining further supported HCH identity. Representative plots are shown, unstained in black and stained in red.

Chondrocytes were highly positive for the cell-lineage markers CD44/73/90 while CD105 resulted weaker, although the complete peak shift with respect to unstained cells suggested the presence of the epitope on the whole population (Figure 1). On the contrary, hemato-endothelial markers CD31/34/45 were not expressed as well as the bone marrow mesenchymal stem cell marker CD271, suggesting the absence of stromal cell contamination in the subchondral bone (Figure 1).

### 2.2. Donors and Treatments Drive Gene Expression Profile

**Figure 2:**
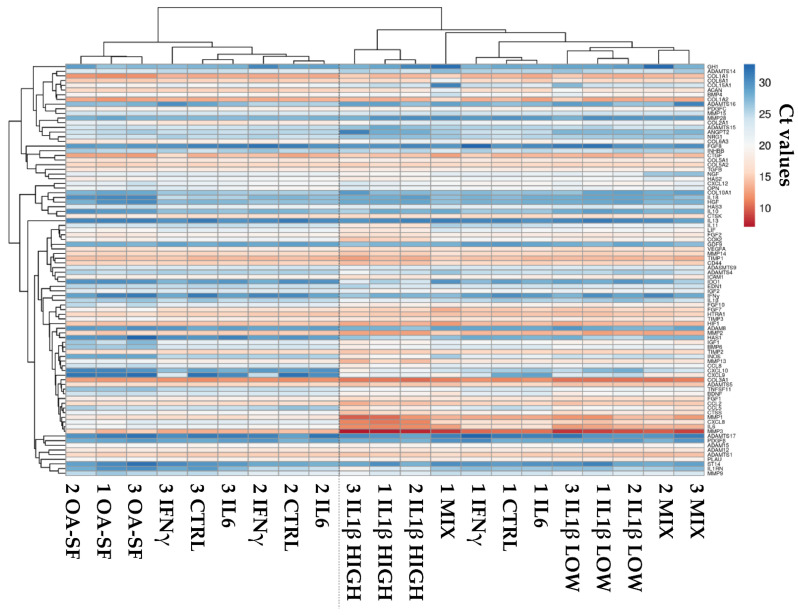
Heatmap showing differential expression of 88 genes between control (CTRL) and stress treatments (OA-SF for synovial fluid from OA-patients, IL1β HIGH for 1 ng/mL IL1β, IL1β LOW for 100 pg/mL IL1β, IL6 for 209 pg/mL IL6, for 86 pg/mL IFNγ and MIX for the three cytokines including IL1β at low levels) in the three HCH donors. Red shades indicate higher expression and blue shades indicate lower expression. Color key indicates the intensity associated with normalized C_t_ values.

Chondrocytes were exposed to naïve OA-SF or one of the five cytokine-based treatments (OA-SF like levels of IL6, IFNγ, IL1β and their combination, and high levels of IL1β). Their RNA was isolated and samples analyzed scoring the expression of 95 genes after normalization with *EF1A*, *RPLP0* and *TBP*. *ADAMTS18*, *CD40LG*, *IL5*, *OSM*, *PLAT*, *TNFα* and *WNT1* not included in the analysis due to their absence or being below the threshold of amplification in the majority of samples. The hierarchical clustering of the whole dataset composed of 88 genes is shown in Figure 2. Without stimuli, resting chondrocytes were distributed in two separate clades, with donors 2 and 3 being more similar in their overall expression profile, due to inter-donor variations as expected for primary cell lines. After stimulation, this situation varied depending on the treatment. IL6-treated samples always clustered tightly to their CTRL, suggesting a negligible effect of this molecule when used at concentrations found in OA-SF. Similarly, IFNγ treatment did not allow the samples to modify their transcriptional pattern, with the treated chondrocytes always under the same clade encompassing CTRL and IL6 ones. This stalemate changed with the addition of IL1β at both OA-SF-like and high concentrations. Low IL1β levels were able to greatly reduce inter-donor variability and create a separate clade. Notably, with the cytokine mix, including OA-SF IL1β levels, samples of donors 2 and 3 appeared similar and under the same clade as those treated with OA-SF IL1β. Regarding donor 1, it was under an intermediate clade covering all samples with low IL1β treatment and other donor 1-related low-impact treatments (CTRL, IL6 and IFNγ). When IL1β was administered at high concentrations, donors clustered showing distance from all those samples with low-level cytokines, including low levels of IL1β. Similarly, OA-SF was able to cluster donors that appeared under a diverging clade with respect to IL1β-related samples but under the same clade encompassing donors 2 and 3 low-impact treatments and control.

**Figure 3:**
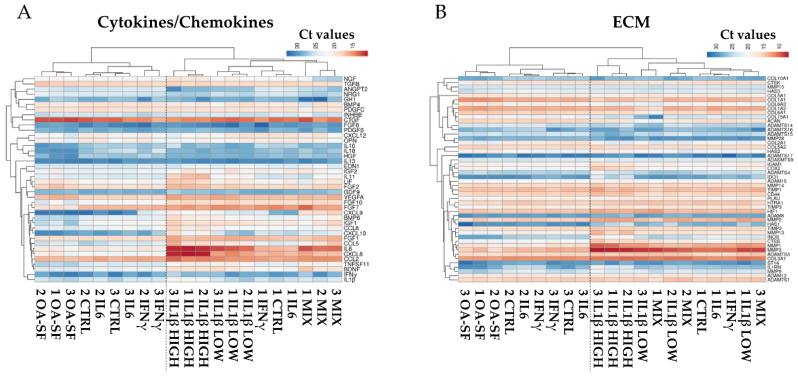
Heatmap showing differential expression of either cytokine/chemokine (**A**) or ECM (**B**) related genes between control (CTRL) and stress treatments (abbreviations as in Figure 2) in the three HCH donors. Red shades indicate higher expression and blue shades indicate lower expression. Color key indicates the intensity associated with normalized C_t_ values.

To obtain further insights about the players able to cluster or separate samples and treatments, the analysis was performed by separating genes depending on their function, either cytokines/chemokines or ECM related. Looking at the first group, a pattern similar to the one observed for the whole dataset emerged (Figure 3A) with the two upper clades encompassing the same samples. The main differences were that IFNγ was able to cluster donors 2 and 3 with respect to CTRL or IL6 and that the mix of low-level cytokines allowed samples to separately lay under the same clade. Regarding ECM, although again the two upper clades confined the same samples, a higher variation emerged (Figure 3B) in the branch described by IL1β-related treatments. High levels of IL1β were still able to group donors, while low IL1β and mixed cytokines did not allow to define distinct clades but resulted in a mixed situation that was not driven by the donors.

### 2.3. IL1β and OA-SF Account for the Strongest Modulation of OA-Related Factors

To identify the players driving the differences between treatments, at first a correlation analysis of the gene expression values were performed. Considering the different conditions separately, OA-SF treatment led to the highest R^2^ value (0.968 ± 0.002), followed by low IL1β (0.956 ± 0.017) and high IL1β (0.954 ± 0.019). As emerged with hierarchical clustering, when not stimulated, donors showed a differential correlation with donor 1 having low similarity with donor 2 (0.902) and 3 (0.913) while the last two behaved similarly (2 vs. 3, 0.962). Although always following the CTRL expression pattern, IL6-treated chondrocytes had a higher overall similarity (0.947 ± 0.021) while a low R^2^ very similar to CTRL emerged for IFNγ (0.921 ± 0.013). Eventually, under treatment with low levels of cytokines, R^2^ was 0.937 ± 0.006, supporting the absence of a unique clade in the dendrogram. To obtain further insights into the analysis, when comparing all the conditions within every single donor, donor 1 had the highest R^2^ value (0.879 ± 0.076), followed by donor 2 (0.848 ± 0.098) and 3 (0.820 ± 0.110). Moreover, when the different comparisons were sifted through donors (Table 1), hierarchical clustering results were again confirmed. The highest R^2^ values emerged for CTRL vs. IL6 (0.984 ± 0.002) and IFNγ (0.959 ± 0.011), and IL6 vs. IFNγ (0.968 ± 0.008). Supporting the idea of IL1β driving the expression pattern when used in combination with the other cytokines, IL1β vs. MIX resulted in 0.927 ± 0.025 while for IL6/IFNγ the comparison was lower (0.798 ± 0.007 and 0.859 ± 0.009, respectively). The impact of IL1β was further confirmed by the high R^2^ between high and low levels of this cytokine (0.908 ± 0.030), and the low correlation with respect to CTRL (0.727 ± 0.091 for high and 0.858 ± 0.060 for low levels). Of note, high IL1β resulted in the condition with the strongest difference with respect to OA-SF (0.657 ± 0.049), suggesting that the two treatments led to a divergent molecular fingerprint. This was confirmed by the high R^2^ between OA-SF and CTRL (0.922 ± 0.026) or IL6 (0.920 ± 0.009). Again, the IL1β effect emerged with low correlation values between OA-SF and both the single cytokine (0.787 ± 0.049) and the cytokine combination (0.702 ± 0.019).

To identify the genes that had shared or differential expression, stability analysis was performed (Table 2 for the top and bottom 10 genes, and Supplementary Table S2 for the whole dataset). In resting conditions, *COL3A1*, *COL5A1* and *TGFB* resulted as the best performers. In OA-SF, *TIMP1*, *MMP15* and *MMP14*. In high levels of IL1β, *ADAMTS5*, *TGFB* and *NRG1*. In low levels of IL1β, *MMP14*, *COL3A1* and *CD44*. For IL6, *CCL2*, *CD44* and *MMP14*. For IFNγ, *COL3A1*, *ADAMTS1* and *ICAM1*. When all low levels of cytokines were used, *CTSK*, *COL5A2* and *TIMP2* appeared. Overall, considering all conditions together, *ADAM15*, *MMP14* and *TIMP2* were the most stable. Therefore, it clearly emerged that, as well as for the overall fingerprint, also single gene stability was dependent on the treatment. Nevertheless, correlation coefficients for single gene stability did not completely mirror what was observed for the overall transcriptional fingerprints (Table 3). If for the crossed correlations between CTRL, IL6 and IFNγ the R^2^ values were the highest ones. The high similarity previously observed between CTRL and OA-SF was lost, ending in a lower and intermediate R^2^, along with the previously described very low correlation value between OA-SF and high IL1β that left the bottom of the group. Interestingly, OA-SF showed a good correlation with IL6, a potentially unexpected result due to the similarity between IL6 and CTRL. Eventually, to better visualize those genes that are always stable between donors regardless of their variations due to treatments, a geometric mean of the single rankings was performed (Supplementary Table S3). *COL3A1*, *MMP14* and *ADAM15* were the best performers suggesting that, independently from the fluctuations in the expression due to the single treatments, these genes have an overall comparable amount between donors.

**Figure 4:**
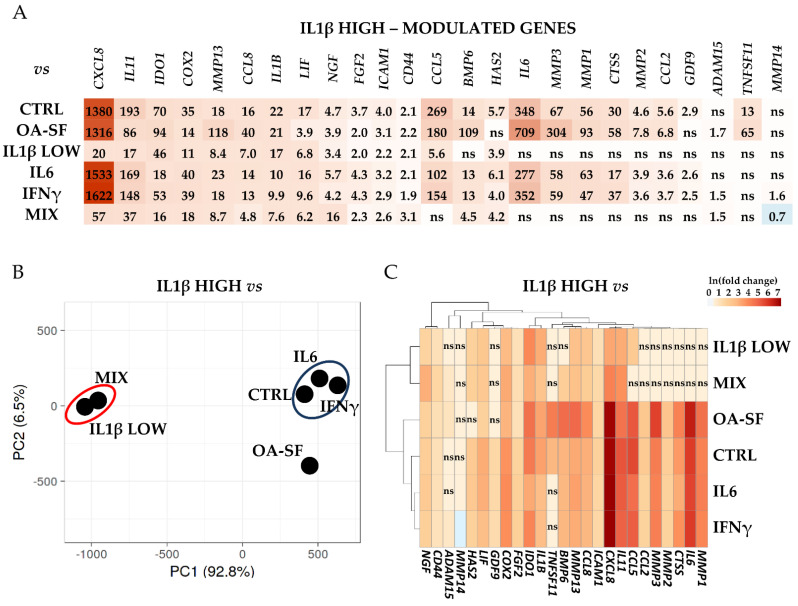
Differential expression of IL1β HIGH-specific genes with respect to control (CTRL) and the other stress treatments (abbreviations as in Figure 2) in the three HCH donors. (**A**) Red shades indicate higher expression ratios and blue shades indicate lower expression ratios of IL1β HIGH with respect to the other conditions. Color key indicates the intensity associated with expression ratios. Mean values are shown. ns = not statistically significant (*p*-value > 0.05). *p*-value for each shown ratio can be found in Supplementary Table S4. (**B**) PCA based on the expression ratios of IL1β HIGH with respect to the other conditions. (**C**) Heatmap based on the ln transformed expression ratios of IL1β HIGH with respect to the other conditions. Red shades indicate higher expression ratios and blue shades indicate lower expression ratios. Color key indicates the intensity associated with expression ratios. ns = not statistically significant (*p*-value > 0.05).

Last, the differential expression for the analyzed genes was calculated. High levels of IL1β allowed for the sharpest response (Figure 4A and Supplementary Table S4 for *p*-values). Twenty-five genes resulted significantly (*p*-value ≤ 0.05) modulated only with this treatment with respect to at least other two conditions. All modulations resulted in an increase in the transcriptional response, with the only exception being a minor downregulation in *MMP14* with respect to the MIX treatment. Within this group, *CXCL8* and *IL11* were the most responsive genes. Notably, the strength of the transcriptional response was often diminished when compared to samples treated with low levels of IL1β (both alone and together with the other cytokines), although in these conditions we did not observe a significant (*p*-value ≤ 0.05) modulation with respect to the other samples. This IL1β-dependent behavior emerged in the principal component analysis (PCA, Figure 4B), where samples containing IL1β at SF-like concentrations (MIX and IL1β low) tightly grouped, clearly separated from CTRL, IL6 and IFNγ-treated samples that gathered in a distinct group not far from OA-SF samples, although more distant. Eventually, hierarchical clustering confirmed that the transcriptional response was directly dependent on IL1β levels for several genes (Figure 4C), including those coding for three metalloproteases (*MMP1/2/3*), three interleukins (*IL6/11* and *CXCL8*), two chemokines (*CCL2/5*) and Cathepsin S (*CTSS*). Of note, IL1β at high levels was able to induce its own transcription, a response that was not observed with low levels of supplementation.

**Figure 5:**
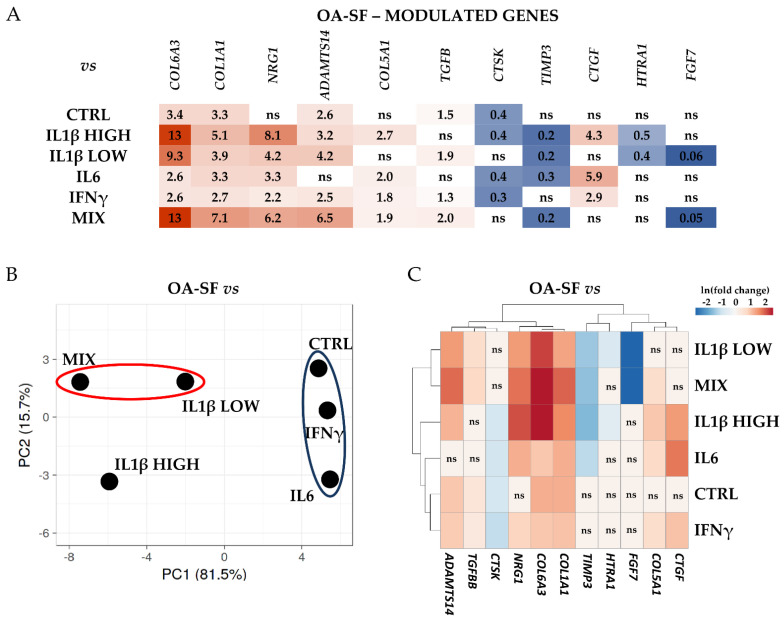
Differential expression of OA-SF-specific genes with respect to control (CTRL) and the other stress treatments (abbreviations as in Figure 2) in the three HCH donors. (**A**) Red shades indicate higher expression ratios and blue shades indicate lower expression ratios of OA-SF with respect to the other conditions. Color key indicates the intensity associated with expression ratios. Mean values are shown. ns = not statistically significant (*p*-value > 0.05). *p*-value for each shown ratio can be found in Supplementary Table S4. (**B**) PCA based on the expression ratios of OA-SF with respect to the other conditions. (**C**) Heatmap based on the ln transformed expression ratios of OA-SF with respect to the other conditions. Red shades indicate higher expression ratios and blue shades indicate lower expression ratios. Color key indicates the intensity associated with expression ratios. ns = not statistically significant (*p*-value > 0.05).

Similarly, although with a reduced extent for both the number of genes and transcriptional modulation, OA-SF specifically drove the amount of 11 genes (Figure 5A and Supplementary Table S4 for *p*-values), that were not altered in any of the other conditions with the exception of *COL5A1* that was upregulated in high levels of IL1β with respect to untreated cells. Two collagen encoding genes (*COL6A3* and *COL1A1*) always resulted in significantly (*p*-value ≤ 0.05) upregulated and most responsive players, with another member of this family (*COL5A1*) having a moderate upregulation in four treatments. Two genes (*NRG1* and *ADAMTS14*) showed an increase in their levels in five conditions out of six. Regarding downregulated players, *CTSK* and *TIMP3* had a reduction in four conditions. From these data, PCA was able to gather samples treated with low levels of IL1β, as well as cluster CTRL, IL6 and IFNγ exposed cells (Figure 5B). Notably, a high amount of IL1β did not drive chondrocytes to cluster with the other samples treated with this factor at a lower concentration. Eventually, hierarchical clustering identified two clades that, differently from the previous analysis, did not separate due to the amount of IL1β (Figure 5C). The main factor that drove the heatmap was the responsiveness with respect to the untreated samples.

**Figure 6:**
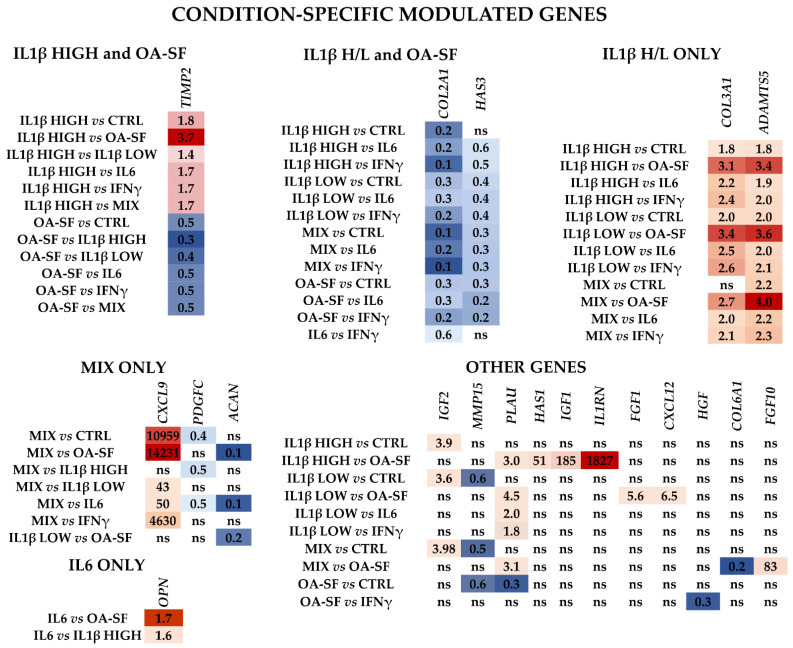
Differential expression of other single or multiple condition specific genes with respect to control (CTRL) and the other stress treatments (abbreviations as in Figure 2) in the three HCH donors. Red shades indicate higher expression ratios and blue shades indicate lower expression ratios of each group/condition with respect to the other conditions. Color key indicates the intensity associated with expression ratios. Mean values are shown. ns = not statistically significant (*p*-value > 0.05). *p*-value for each shown ratio can be found in Supplementary Table S4.

No obvious influence given by a specific condition could be identified regarding the other genes (Figure 6 and Supplementary Table S4 for *p*-values). *TIMP2* was regulated by both high IL1β and OA-SF with a diverging pattern (moderate upregulation for the cytokine and downregulation for the synovial fluid). *COL2A1* and *HAS3* were the only two genes regulated by both concentrations of IL1β (including the MIX) and OA-SF and were always downregulated with the only exception of high IL1β vs. CTRL. Interestingly, two genes were uniquely modulated by IL1β, regardless of its concentration or presence of other cytokines (MIX). Both *COL3A1* and *ADAMTS5* were always significantly upregulated, except for MIX versus control cells for collagen (ratio of 1.6, *p*-value of 0.0806), with the strongest increase when compared to OA-SF. Three genes responded to the mix of low-level cytokines, with *PDGFC* and *ACAN* reduced in their amount and *CXCL9* being strongly upregulated, with the ratio with high IL1β (7.4) that almost reached significance (*p*-value of 0.0517). Sifting the other components of the MIX condition, only one gene (*OPN*) seemed to respond to IL6, although with low impact. No specific genes were found for IFNγ. Eventually, a few other genes had a response that could not be attributed to a specific treatment, although often this was regulated by IL1β or OA-SF.

### 2.4. Validation of Identified IL1β High and OA-SF-Modulated Genes

**Figure 7:**
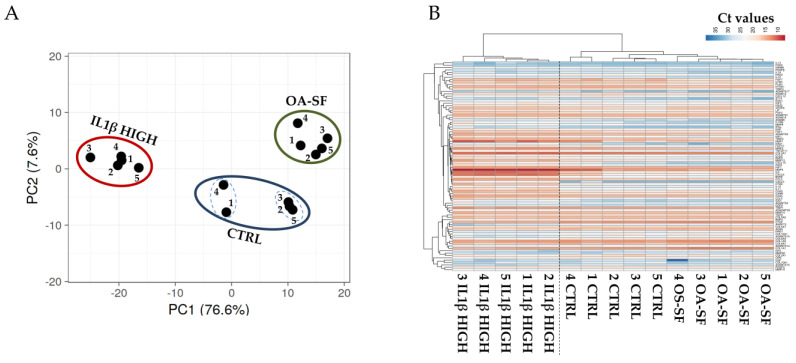
PCA (**A**) and heatmap (**B**) showing differential expression of 88 genes between control (CTRL) and stress treatments (abbreviations in Figure 2) in the previously analyzed three HCH donors (1/2/3) and two new independent ones (4/5). In the heatmap, red shades indicate higher expression and blue shades indicate lower expression. Color key indicates the intensity associated with normalized C_t_ values.

**Figure 8:**
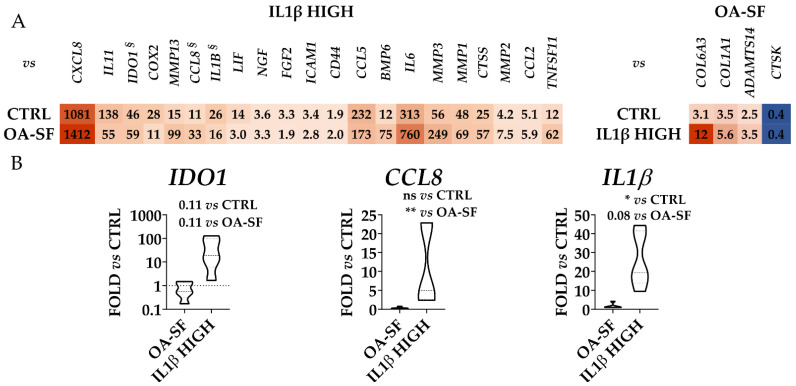
Differential expression of IL1β HIGH and OA-SF-specific genes (only those significantly modulated vs. each other in Figure 4 and Figure 5) with respect to control (CTRL) and the other treatment for the five HCH donors. (**A**) Red shades indicate higher expression ratios and blue shades indicate lower expression ratios with respect to the other conditions. Color key indicates the intensity associated with expression ratios. Mean values are shown. *p*-value for each shown ratio can be found in Supplementary Table S5. (**B**) Violin plots show the expression of the genes with *p*-values > 0.05 in one or both ratios (§ in panel A). * for *p*-value ≤ 0.05, ** for *p*-value ≤ 0.01.

To validate the gene expression patterns that resulted strongly dependent on high levels of IL1β or OA-SF, two additional donors (donors 4 and 5) were analyzed. Additionally, for these donors, flow cytometry analysis confirmed the presence of high levels of CD44/73/90, at moderate levels of CD105 and the absence of CD31/34/45/271. From PCA and hierarchical clustering, it clearly emerged that, for each condition, these donors tightly clustered with their homologs (Figure 7A,B). Moreover, as previously shown, untreated samples resulted in more heterogeneity and could be divided into two smaller subgroups (samples 1-4 and 2-3-5), while after treatments the gene expression patterns became more similar. Notably, again confirming the differential expression data for the previous three donors, high levels of IL1β were able to modulate with a stronger impact on the gene transcripts leading to the coexistence of untreated and OA-SF samples under a separate and shared clade in the heatmap. To obtain further insights into the donor-unrelated conservation of gene modulation given by the two treatments, the most diverging genes (significant difference with both CTRL and the other condition) previously identified were analyzed (Figure 8 and Supplementary Table S5 for *p*-values). Out of 25 genes, 22 confirmed the differential expression vs. both untreated cells and the other condition, with these last samples being non-statistically different (Figure 8A). The only gene that did not show a significant (*p*-value ≤ 0.05) difference nor tendency (*p*-value ≤ 0.1) was the IL1β-sensitive *IDO1*, although in all donors the cytokine treatment led to an increase in its expression with respect to the other conditions (*p*-value of 0.11 for both due to high heterogeneity of modulation) (Figure 8B). The other two IL1β-modulated genes presented an intermediate situation. *CCL8* was significantly (*p*-value ≤ 0.01) different only vs. OA-SF although, as before, IL1β was able to promote its upregulation with respect to CTRL in all donors (Figure 8B). *IL1β* itself was significantly (*p*-value ≤ 0.05) different only vs. untreated cells, with a tendency (*p*-value of 0.0806) towards OA-SF (Figure 8B). Therefore, the modulation previously observed was confirmed in the two additional donors.

## 3. Discussion

In this work, the transcriptional response of chondrocytes to conditions mimicking in vitro the OA pathology was analyzed and compared. Out of a panel of genes coding for several factors involved in OA initiation and progression, the results of this study clearly indicated that synovial fluid from patients and the most commonly used IL1β treatment lead to a divergent molecular response, opening the question of whether high levels of inflammation may effectively recapitulate OA in vitro.

For decades, OA has been considered a degenerative chronic disease mostly characterized by cartilage degradation. In the last years, basic research and clinical evidence have indicated that all the articular tissues are affected by and at the same time drive OA progression [18]. In particular, the initial cartilage degradation in the early stages of OA results in tissue particles, debridement and microcrystals that enter the synovial fluid. These components are phagocytosed by synovial macrophages which trigger the inflammatory process [19] through the synthesis of pro-inflammatory cytokines and matrix-degrading enzymes [20] which, in turn, diffuse through the synovial fluid into the cartilage generating a vicious circle. At the end of this process, cartilage is progressively degraded thus sustaining and worsening the condition by producing additional inflammation [21]. Therefore, inflammation has been envisioned both as a landmark of OA pathology and as a methodological approach to mimic the OA phenotype in vitro, especially when studying cartilage and chondrocyte response. Among the most studied molecules, IL1β, IL6, IFNγ and TNFα have been intensively investigated.

In the pooled synovial fluid used in this study, the presence of TNFα was not detected [22]. This is in agreement with other reports where this cytokine was not identified or often reported to be in a range (1–10 pg/mL) [23] close to the inferior detection limit of the assay used here (4 pg/mL, with the ELISA analysis performed on a 50% diluted sample). For this reason, this molecule was not included in the analysis, we choose to focus on cytokines detected in the 100–200 pg/mL range such as IL1β, IL6 and IFNγ. Accordingly, in several reports, these molecules were used to trigger an inflammatory state [24], with IL1β being by far the most described. In the 1–10 ng/mL range, this cytokine was revealed to induce inflammation mediated by *IL6* synthesis [25], increase gene expression and protein release of pro-inflammatory cytokines, chemokines and MMPs in cells [26] and cartilage explants [27], and inhibit type II collagen and proteoglycans synthesis [28]. Of note, the same transcriptional pattern was observed in this study, with high levels (1 ng/mL) of IL1β able to upregulate the transcription of several metalloproteases (*MMP1/2/3/13*), inflammatory cytokines (*CXCL8*, *IL1β/6/11*) and CC chemokines that are chemotactic for monocytes and lymphocytes and exert inflammatory functions (*CCL2/5/8*). Moreover, *COL2A1* was downregulated, suggesting that the protocol and chondrocytes used in the study were consistent with the literature.

The main drawbacks of this treatment are that the administered concentration was 10 times higher than the levels observed in OA patients’ synovial fluid and that the synovial fluid itself is a blend of hundreds of molecules, making the effects of a single cytokine an oversimplified view. To stick more closely to the physiological condition, the effect of IL1β, IL6, IFNγ and the combination of these three molecules was studied at synovial fluid-like concentrations. Hierarchical clustering, principal components, correlation and gene expression analyses clearly showed that IL6 and IFNγ were not able to influence chondrocyte transcriptional profile. Their main effect was exerted on *CXCL9*, when their combination with low levels of IL1β was able to increase its transcription with respect to the single molecules. IL1β was able to alter chondrocyte gene expression also in conditions similar to those found in patients’ synovial fluid. This is clearly shown by the heatmaps and PCA where a separate clade encompassing all donors emerged, suggesting a reduction in the inter-donor variability detected in untreated or IL6/IFNγ treated cells where the three donors lay under separate clades. This is in agreement with the recently reported abolishment of donor-dependent variations in immune modulatory function in mesenchymal stromal cells treated with inflammatory cytokines [29]. This outcome might be of importance for physiology studies framed by specific subsets of factors, since differences between donors, especially when analyzing few samples, may hinder a condition-dependent response or phenotype. Nevertheless, at a single gene expression level the response was greatly reduced with respect to the one observed for high levels of this cytokine. A striking example is *IL1β* itself, which resulted in upregulation only at the 1 ng/mL concentration, as well as for the genes in Figure 4. This is further emphasized by the heatmap where samples treated with synovial fluid levels of IL1β, both alone or in combination with the other cytokines, clearly separate from those in high concentrations of IL1β. Altogether, these results showed that not all cytokines at synovial fluid-like levels have an effect on chondrocytes in vitro and that IL1β is the most potent molecule able to polarize cell transcriptional patterns in a dose-dependent manner. Therefore, it may be presumable that synovial fluid, due to its heterogeneous composition, may be the result of the combinatorial effects of factors having a humble influence per se, with others having only a negligible role as we observed for IL6 and IFNγ that were not able to boost the outcomes of low IL1β.

Under this paradigm, the crucial need emerges to deeply characterize the synovial fluid used in the experiments to evaluate the weight of single or multiple factors and the overall impact. This is the reason why we opted for a pooled OA patients’ synovial fluid batch that was thoroughly sifted for 200 factors [22], including the previously described IL1β, IL6 and IFNγ. In the literature, few examples of chondrocytes treated with OA-SF are present, albeit with contrasting results. As an example, Hoff and colleagues showed the increment of IL6 and CXCL8 after 24/48 h of treatment [30] while Housmans and colleagues in the same timeframe (24/72 h) recorded a reduction in gene expression [31]. Despite differences in the detection method, in both cases, no information regarding synovial fluid composition was reported making a direct comparison between them and this study difficult. Here, we could clearly show that OA-SF was able to polarize donors divergent from the outcomes observed with low and especially high levels of IL1β, with samples sharply separated in both PCA and hierarchical clustering analyses. Only a few genes were shared, *COL2A1* and *HAS3* resulted in downregulation in both conditions. The reduction in the expression of *HAS3* could contribute to the lower levels of hyaluronic acid concentration observed in OA patients’ synovial fluid, although this molecule is mainly secreted by synovial fibroblasts [32]. Additionally, the effect on *COL2A1* transcription supports the damage in collagen II structure observed in both superficial/upper mid and lower mid/deep zones of the OA cartilage, with the initial damage always seen around chondrocytes [33] that are not able to efficiently synthesize the polymer. On the contrary, *TIMP2* had a divergent modulation, being moderately upregulated with IL1β and downregulated with OA-SF stimulation. This pattern might be part of an opposite regulation ending in a similar effect on the ECM. In fact, IL1β, together with only *TIMP2* as a protective mechanism strongly upregulated destructive metalloproteases such as *MMP1/2/3/13*, while OA-SF led to a reduction in *TIMP2* and *3*. Therefore, in both cases, a similar misbalance in the MMPs/TIMPs ratio, together with the reduction in collagen II levels, could result in the destabilization of the cartilage ECM. Moreover, with OA-SF, a general effect on other collagen types was observed. If *COL2A1* was reduced, *COL6A3*, *COL1A1* and *COL5A1* increased their transcription. Therefore, OA-SF affects the ECM not only through the degradation of cartilaginous collagen (type II) but also by producing fibrous collagens (type I and VI, and V that occurs as heterotypic fibrils with type I). Consistently, in patients with OA, both *COL1A1* [34] and *COL6A3* [35] have been reported to be upregulated. This mechanism acting on collagens was less activated by IL1β, where only the downregulation of *COL2A1* and upregulation of the fibrous collagen encoding gene *COL3A1* emerged. Eventually, the last difference between IL1β and OA-SF effects was the lack of modulation of inflammation-related genes, such as the cytokines *CXCL8*, *IL1β/6/11* and the chemokines *CCL2/5/8*, in synovial fluid treated samples. It is presumable that OA-SF contributes to joint inflammation to a lesser extent and indirectly through the generation of collagen micro fragments able to trigger an inflammatory response in synovial macrophages, rather than directly through the massive secretion of pro-inflammatory mediators from chondrocytes as observed with high levels of IL1β. Overall, IL1β and OA-SF promote cartilage ECM turnover and OA progression through a partially overlapping mechanism acting on collagen, biased towards the increased degradation by proteases for IL1β and towards fibrous replacement for OA-SF. Eventually, the main difference between treatments is the lack of a direct inflammatory response given by the synovial fluid, also demonstrated by the lack of upregulation for inflammation-related *COX2* [36], *IDO1* [37] and *LIF* [38]. This is consistent with the very reduced (low IL1β) or absent (IL6 and IFNγ) response to cytokines, even when in combination at SF-like concentrations. Therefore, IL1β and OA-SF act on different pathways and molecules that, although resulting in similar overall phenotypes for ECM homeostasis, are only partly super-imposable, opening the question of whether high levels of inflammation might mimic OA at a molecular level in vitro.

We are aware that this work has some limitations. First, chondrocytes were obtained from the intact part of cartilage from OA patients due to the impossibility to obtain cartilage biopsies from healthy patients for ethical reasons. Although our experiments with IL1β gave results in agreement with the literature on their responsiveness, even when used at low concentrations indicating fully responsive cells such as those obtained from “healthy” cartilage, a possibly reduced cell sensitivity to OA synovial fluid or inflammatory cytokines at OA-like concentrations cannot be excluded. Additionally, protocols based on cytokine stimulation are usually performed in a short time frame (24–72 h), as we conducted for all the treatments including OA-SF, while the cartilage phenotype in OA patients is the result of years of an evolving pathology. Third, we opted for a pooled synovial fluid batch. Although reducing the donor-dependent differences and the more specific interaction given by donor-matched chondrocytes and synovial fluid, this choice allowed us to obtain enough volume to perform both a thorough characterization of its composition and the experiments herein described. Eventually, molecular results presented here will need further validation at the protein level, at least for the most distinctive players. As an initial confirmation of the gene expression data proposed, the results obtained at the protein level on a different set of chondrocytes in a previous publication of our group showed that IL1β at 1 ng/mL was able to induce IL6 release while no modulation of IL1RN emerged [39].

## 4. Materials and Methods

### 4.1. Isolation and Expansion of Human Articular Chondrocytes

Human articular chondrocytes were obtained by enzymatic digestion of intact portions of the articular cartilage collected from five women who underwent total knee arthroplasty (mean age 67 yo of ± 2 for donors 1/2/3 and 69 yo ± 6 for donors 4/5, overall 68 yo ± 4) and affected by osteoarthritis of Kellgren-Lawrence grade III-IV. Briefly, the cartilage was digested with 0.15% (*w*/*v*) Type II Collagenase (Worthington Biochemical Corporation, Lakewood, NJ, USA), for 22 h at 37 °C under shaking. Released chondrocytes were immediately frozen in liquid nitrogen. At the time of experiments, chondrocytes were thawed and seeded at a density of 1 × 10^4^ cells/cm^2^ in high glucose Dulbecco’s modified Eagle medium supplemented with 10 % (*v*/*v*) FBS, 1 % (*v*/*v*) 200 mM L-Glutamine, 1% 10,000 U/mL Penicillin, 10 mg/mL Streptomycin and 1 % (*v*/*v*) 250 µg/mL Amphotericin B (all reagents from Thermofisher Scientific, Waltham, MA, USA) and incubated at 37 °C, 5 % CO_2_ until the 80% confluence was reached. After cell detachment, one aliquot was used for flow cytometry analysis and another aliquot was seeded at 3 × 10^4^ cells/cm^2^ to perform the experiments avoiding the dedifferentiation given by prolonged passaging.

### 4.2. Flow Cytometry

Cells in suspension were stained for 30 min at 4 °C in the dark with the following antibodies: chondrocyte (CD44-PE Vio770 clone REA690, CD73-PE clone REA804, CD90-FITC clone REA897, CD105-PerCP Vio700 clone REA794) markers, hemato/endothelial (CD31-PerCP Vio700 clone REA730, CD34-FITC clone AC136, CD45-PE Vio770 clone REA747) markers and bone marrow mesenchymal stromal cell (CD271-PE clone REA844) markers (all from Miltenyi Biotec, Bergisch Gladbach, Germany). After the cell wash with FACS buffer (phosphate buffer, 5% FBS, 0.1% sodium azide), cells were detected by flow cytometry using a CytoFLEX flow cytometer (Beckman Coulter, Fullerton, CA, USA). The following antibody combinations were used: CD73/90/105/44 and CD34/271/31/45 (FITC, PE, PC5 and PC7 channels, respectively, for both combinations).

### 4.3. Cell Treatments

Twenty-four hours after seeding, cells were cultured for 72 h as either untreated (in complete medium, condition named as CTRL) or treated with: a pool of synovial fluids, obtained from 13 osteoarthritis patients (eight females, five males, mean age 69 yo ± 8; Kellgren-Lawrence grade III–IV) undergoing total knee arthroplasty (100% synovial fluid with no addition of complete medium, OA-SF) centrifuged at 16,000× *g*, 10 min at RT to remove floating cells and debris, with no addition of medium or serum to mimic the joint cavity environment; IL1β at 1 ng/mL in complete medium (IL1β high) as standard concentration to induce OA phenotype in vitro; IL1β at 100 pg/mL (IL1β low); IL6 at 209 pg/mL in complete medium (IL6); IFNγ at 86 pg/mL in complete medium (IFNγ); or a mix including the three cytokines (IL1β low, IL6 and IFNγ) in complete medium (MIX). Recombinant cytokines were purchased from PeproTech (Rocky Hill, NJ, USA). The concentration of IL1β low, IL6 and IFNγ was retrieved from the biochemical characterization of the pooled synovial fluid used for the condition OA-SF, which was previously performed in our laboratory [22]. In this analysis, no TNFα was detected, and therefore, this cytokine was not used further. Donors 1/2/3 received all treatments, while donors 4/5 received CTRL, OA-SF and IL1β high treatments. After incubation for 72 h, an equal volume of hyaluronidase solution (2 mg/mL in phosphate buffer; Sigma Aldrich, St. Louis, MO, USA) was added for 2 min at 37 °C under shaking to each well. This was conducted to render the viscous synovial fluid easier to be removed in the OA-SF samples, and in the other conditions characterized by complete medium-based formulations, it was performed to avoid differential treatments with respect to synovial fluid-treated cells. After supernatant removal, cells were lysed by directly adding the RLT solution of the RNeasy Mini Kit (Qiagen, Hilden, Germany), and the suspension stored at −80 °C.

### 4.4. RNA Isolation and cDNA Synthesis

Cell lysates were thawed and RNA was purified with the RNeasy Mini Kit (Qiagen) following the modified manufacturer’s protocol for contaminating gDNA removal. After quantification of the purified RNA, 50 ng were retrotranscribed with the iScript kit (Bio-Rad Laboratories, Hercules, CA, USA) and cDNA was pre-amplified with SsoAdvanced PreAmp Supermix kit (BioRad Laboratories) according to the protocol provided by the manufacturer. Afterward, cDNA was diluted to a final volume of 1 mL with DNase-free water and frozen for future use at −20 °C.

### 4.5. Gene Expression

Primers (Supplementary Table S1) were designed in-house with the NCBI Primer Designing Tool (http://www.ncbi.nlm.nih.gov/tools/primer-blast/, accessed on 19 July 2022) in order to recognize the largest number of known splice variants. When possible, one primer for each couple had to span an exon-exon junction. Gene expression was evaluated with iTaq™ Universal SYBR^®^ Green Supermix (BioRad), following the manufacturer’s instructions, using the QuantStudio™ Real-Time PCR system (Thermofisher Scientific). Each sample was analyzed in technical triplicate. Amplification for each gene was considered present when the majority of calls resulted in lower than 30 C_t_. *RPLP0*, *EF1α* and *TBP* were used as housekeeping genes for data normalization between treatments.

### 4.6. Computational Anlyses

Principal component analysis (PCA) and hierarchical clustering were obtained with the ClustVis package (https://biit.cs.ut.ee/clustvis/, accessed on 19 July 2022) [40]. Maps were generated using the following settings: no transformation for Ct values after housekeeping normalization or ln(x) transformation for fold change in gene expression; no row centering; no unit variance scaling; PCA method: SVD with imputation.

### 4.7. Statistical Analysis

Statistical analysis was performed using GraphPad Prism Software version 8.0.2 (GraphPad, San Diego, CA, US). Normal data distribution was assessed by the Shapiro–Wilk normality test (α of 0.01). When the normality test was passed, a repeated measures one-way ANOVA test was performed, with Tukey’s post-hoc test. When the normality test was not passed, a nonparametric Friedman test was executed, with Dunn’s post-hoc test. The level of significance was set at *p*-value ≤ 0.05.

## 5. Conclusions

High levels of IL1β and synovial fluid from OA patients are able to polarize the gene expression profile of chondrocytes in vitro. Our data showed that, despite having a similar influence on ECM homeostasis, the pathways activated are divergent, with IL1β mainly acting on proteases and the synovial fluid altering collagen composition. Moreover, synovial fluid was not able to induce the inflammatory response elicited by IL1β. These results open the question of whether, although with partially overlapping phenotypes, acute inflammation-based protocols can efficiently mimic the OA phenotype for molecular studies in vitro.

## Figures and Tables

**Table 1:** Correlation analysis for gene expression profiles of control and treated chondrocytes.

	CTRL	OA-SF	IL1β HIGH	IL1β LOW	IL6	IFNγ	MIX
CTRL		**0.922 _± 0.026_**	**0.727 _± 0.091_**	**0.858 _± 0.060_**	**0.984 _± 0.002_**	**0.959 _± 0.011_**	**0.799 _± 0.039_**
OA-SF			**0.657 _± 0.049_**	**0.787 _± 0.049_**	**0.920 _± 0.009_**	**0.882 _± 0.023_**	**0.702 _± 0.019_**
IL1β HIGH				**0.908 _± 0.030_**	**0.746 _± 0.080_**	**0.779 _± 0.076_**	**0.879 _± 0.013_**
IL1β LOW					**0.874 _± 0.051_**	**0.891 _± 0.043_**	**0.927 _± 0.025_**
IL6						**0.968 _± 0.008_**	**0.798 _± 0.007_**
IFNγ							**0.859 _± 0.009_**
MIX							

Values indicate mean R^2^ with standard deviation. Background color shade indicates degree of correlation from red (lower correlation) to green (higher correlation).

**Table 2:** Top 10 and bottom 10 ranking positions in the stability between donors for the expression of genes in each experimental condition.

Rank	CTRL	OA-SF	IL1β HIGH	IL1β LOW	IL6	IFNγ	MIX	All Samples
**1**	*COL3A1*	*TIMP1*	*ADAMTS5*	*MMP14*	*CCL2*	*COL3A1*	*CTSK*	*ADAM15*
**2**	*COL5A1*	*MMP15*	*TGFB*	*COL3A1*	*CD44*	*ADAMTS1*	*COL5A2*	*MMP14*
**3**	*TGFB*	*MMP14*	*NRG1*	*CD44*	*MMP14*	*ICAM1*	*TIMP2*	*TIMP2*
**4**	*NRG1*	*ADAM15*	*ADAMTS1*	*TIMP1*	*ADAM15*	*NRG1*	*CCL2*	*COL5A2*
**5**	*CTSK*	*TNFSF11*	*CD44*	*PDGFC*	*GDF9*	*TIMP2*	*HAS3*	*TIMP1*
**6**	*PLAU*	*NGF*	*ADAM15*	*ANGPT2*	*COL1A1*	*ADAM15*	*TGFB*	*ADAM12*
**7**	*ADAMTS5*	*FGF1*	*HIF1*	*TIMP2*	*COL6A1*	*COL5A1*	*ADAMTS5*	*ADAMTS1*
**8**	*CCL2*	*CTSS*	*PDGFC*	*TGFB*	*TNFSF11*	*COL5A2*	*FGF10*	*COL3A1*
**9**	*CD44*	*COL6A1*	*BDNF*	*MMP13*	*TIMP2*	*PLAU*	*MMP13*	*HTRA1*
**10**	*IL13*	*BMP6*	*TIMP2*	*FGF1*	*TIMP1*	*HIF1*	*ADAM15*	*CD44*
**79**	*MMP28*	*MMP3*	*HAS1*	*ADAMTS16*	*HAS1*	*IL1RN*	*HAS2*	*MMP1*
**80**	*CXCL9*	*HGF*	*COL2A1*	*IGF2*	*IL18*	*MMP3*	*IDO1*	*IL11*
**81**	*FGF10*	*CXCL8*	*EDN1*	*COL10A1*	*IGF2*	*COL15A1*	*ADAMTS16*	*INOS*
**82**	*MMP3*	*GH1*	*COL10A1*	*COL6A1*	*CXCL10*	*IL6*	*ACAN*	*MMP3*
**83**	*COL2A1*	*IGF2*	*IDO1*	*ST14*	*MMP3*	*GH1*	*IL11*	*CXCL10*
**84**	*IL6*	*CXCL12*	*IGF2*	*ACAN*	*COL2A1*	*COL2A1*	*GH1*	*COL15A1*
**85**	*IL1RN*	*ST14*	*CXCL10*	*CXCL10*	*IL6*	*CXCL10*	*COL6A1*	*CCL5*
**86**	*MMP1*	*HAS1*	*ANGPT2*	*HAS1*	*MMP1*	*ADAMTS16*	*NGF*	*IL6*
**87**	*COL15A1*	*FGF10*	*ADAMTS4*	*BMP4*	*INOS*	*MMP1*	*BMP4*	*CXCL8*
**88**	*CXCL8*	*COL2A1*	*IL1RN*	*COL15A1*	*CXCL8*	*CXCL8*	*COL15A1*	*CXCL9*

**Table 3:** Correlation analysis of the stability rankings for single gene expression of control and treated chondrocytes.

	CTRL	OA-SF	IL1β HIGH	IL1β LOW	IL6	IFNγ	MIX
CTRL		**0.198**	**0.223**	**0.361**	**0.414**	**0.610**	**0.124**
OA-SF			**0.178**	**0.111**	**0.354**	**0.293**	**0.029**
IL1β HIGH				**0.229**	**0.260**	**0.283**	**0.100**
IL1β LOW					**0.177**	**0.295**	**0.179**
IL6						**0.543**	**0.100**
IFNγ							**0.155**
MIX							

Values indicate R^2^. Background color shade indicates degree of correlation from red (lower correlation) to green (higher correlation).

## Data Availability

Raw data for this study are available at https://osf.io/zea3h/?view_only=9dce4358c8b74a328b3beef6c0625519, created on 25 July 2022.

## Supplementary Materials

The following supporting information can be downloaded at: https://www.mdpi.com/article/10.3390/ijms24032625/s1, Table S1: List of primers used in the study; Table S2: Ranking positions in the stability between donors for the expression of genes in each experimental condition: Table S3: Ranking positions for the stability of genes between donors regardless the treatment-related fluctuations; Table S4: *p*-values of condition-specific regulated genes; Table S5: *p*-values of IL1β high or OA-SF-specific regulated genes.
